# A Comparative Study of Mesoporous Silica and Mesoporous Bioactive Glass Nanoparticles as Non-Viral MicroRNA Vectors for Osteogenesis

**DOI:** 10.3390/pharmaceutics14112302

**Published:** 2022-10-26

**Authors:** Sepanta Hosseinpour, Maria Natividad Gomez-Cerezo, Yuxue Cao, Chang Lei, Huan Dai, Laurence J. Walsh, Saso Ivanovski, Chun Xu

**Affiliations:** 1 School of Dentistry, The University of Queensland, Herston, QLD 4006, Australia; 2 Departamento de Química en Ciencias Farmacéuticas, Facultad de Farmacia, Universidad Complutense de Madrid, Instituto de Investigación Sanitaria Hospital 12 de Octubre i+12, Plaza Ramón y Cajal s/n, 28040 Madrid, Spain; 3 The Pharmacy Australia Centre of Excellence (PACE), The University of Queensland, Woolloongabba, QLD 4102, Australia; 4 Australian Institute for Bioengineering and Nanotechnology, The University of Queensland, St Lucia, QLD 4072, Australia

**Keywords:** gene delivery, mesoporous silica nanoparticle, mesoporous bioactive glass nanoparticle, microRNA therapy, bone regeneration

## Abstract

Micro-ribonucleic acid (miRNA)-based therapies show advantages for bone regeneration but need efficient intracellular delivery methods. Inorganic nanoparticles such as mesoporous bioactive glass nanoparticles (MBGN) and mesoporous silica nanoparticles (MSN) have received growing interest in the intracellular delivery of nucleic acids. This study explores the capacity of MBGN and MSN for delivering miRNA to bone marrow mesenchymal stem cells (BMSC) for bone regenerative purposes, with a focus on comparing the two in terms of cell viability, transfection efficiency, and osteogenic actions. Spherical MBGN and MSN with a particle size of ~200 nm and small-sized mesopores were prepared using the sol-gel method, and then the surface was modified with polyethyleneimine for miRNA loading and delivery. The results showed miRNA can be loaded into both nanoparticles within 2 h and was released sustainedly for up to 3 days. Confocal laser scanning microscopy and flow cytometry analysis indicated a high transfection efficiency (>64%) of both nanoparticles without statistical difference. Compared with MSN, MBGN showed stronger activation of alkaline phosphatase and activation of osteocalcin genes. This translated to a greater osteogenic effect of MBGN on BMSC, with Alizarin red staining showing greater mineralization compared with the MSN group. These findings show the potential for MBGN to be used in bone tissue engineering.

## 1. Introduction

A large variety of ribonucleic acid (RNA)-based therapies for enhancing bone regeneration have been developed, including microRNA (miRNA) [1], messenger RNA [2], and small interfering RNA [3]. For use in regenerative medicine, miRNA may overcome the limitations of other methods, particularly the use of directly delivered growth factors and plasmid DNA [4,5,6,7]. A wide variety of miRNAs orchestrate osteoblast and osteoclast differentiation, and influence osteogenesis signalling pathways as well as osteogenic transcription factors [8,9]. Several miRNA dysregulations have been found in bone disorders such as osteoporosis, osteopetrosis, osteogenesis imperfecta, and osseous malignancies [10,11].

Major advantages of using miRNAs include very low working doses, sustained expression of genes, and prolonged regulatory activity [4]. The cytoplasmic half-life of miRNA extends from one to several days [5], which exceeds that of growth factors (several hours) [7]. miRNAs have a favourable balance of efficiency versus cost when compared to plasmid DNA or growth factors [4,12].

Despite these advantages, the delivery of plain microRNA remains challenging because of degradation by extracellular enzymes, their high negative charge, and problems crossing the cell membrane barrier [13]. These issues led to the use of viral vectors as delivery systems for transfecting cells [14]; however, this approach has limitations including limited loading capacity, high cost, and concerns about immunogenicity and mutagenicity [15].

As an alternative, synthetic nanoparticles have gained growing attention [16]. Nanoparticles are capable of efficiently transfecting cells with low toxicity. Among the various types of nano-vectors, mesoporous silica nanoparticles (MSN) have been used to deliver drugs, proteins, and nucleic acids [17,18,19,20,21]. They have a large surface area, tunable pore and particle size, and high biocompatibility [22,23,24]. Their surface can be functionalized, improving their delivery potential for nucleic acids including plasmid DNA and various types of RNA [25,26]. Mesoporous bioactive glass nanoparticles (MBGN) are inorganic nanoparticles with the composition of silica, calcium, phosphate and/or other elements and display therapeutic potential for bone regeneration [27,28,29]. While the mesoporous properties of MBGN are similar to those of MSN, their major advantage is greater bioactivity for promoting bone formation due to calcium ion release [30,31]. MBGN can be used to deliver drugs and nucleic acids [32,33,34,35,36]. Release of the incorporated calcium ions within the glass structure gives MBGN a higher biological activity in bone regeneration [37,38]. Compared to other bone repairing materials such as hydroxyapatite, beta-tricalcium phosphate (β-TCP), the porous nature of MSN and MBGN give them the advantages of large drug loading capacity, gene delivery ability and higher bioactivities. In addition, the particles and pore structure can be carefully tuned to meet the requirements [22,23,24].

With super osteogenesis alibility and porous nature, MBGN has been applied widely for bone regeneration and drug delivery [39,40]. Previously, applications of MBGN are mainly focused on the design as bone-filling materials or scaffolds, and the loaded drugs are mainly small molecular drugs [37,38]. With the growing interest in miRNA therapy, there are very few reports using MBGN for genes, especially miRNA delivery. In addition, despite both MSN and MBGN being promising as nano-vectors for gene therapy for the treatment of bone defects, there is no report to compare the miRNA delivery efficacy for bone repairing to the best of our knowledge.

In this study, we prepare surface functionalized spherical MSN and MBGN with similar particle size and small pore size (<10 nm) and evaluate the miRNA delivery efficiency towards BMSC. We compare the miRNA loading capacity and release profile, as well as the cellular toxicity and cellular update efficacy. Then, we use both nanoparticles to deliver a functional miRNA (miRNA-26a) and compared the osteogenesis functionality towards BMSCs. This study can provide guidance for the design of functional MBGN and MSN for functional miRNA delivery in bone tissue engineering.

## 2. Materials and Methods

### 2.1. Synthesis and Surface Modifications of Nanoparticles

MSN synthesis: MCM-41-type MSN was synthesized using a previously described synthesis technique [21]. In brief, 0.2 g of cetyltrimethylammonium bromide (CTAB, Sigma, St. Louis, MO, USA) was dissolved in 96 g of deionized water with stirring at room temperature, and then 0.7 mL NaOH solution (2 M) was added and stirred for about 20 min until CTAB powder was dissolved. Then, 1.43 mL tetraethyl orthosilicate (TEOS, Sigma, Clayton, Australia) was added to the above solution and the mixture was stirred for 2 h at 80 °C. High-speed centrifugation has been used to separate the products and then calcined at 550 °C for 5 h.

MBGN synthesis: The synthesis of MBGN was performed following the previously reported methods [28,41]. Shortly after, two different solutions were prepared: (1) poly(styrene)-block-poly(acrylic acid) (PS-b-PAA) in tetrahydrofurane (THF) at room temperature and (2) CTAB in basic deionized water with ammonia (28% *w*/*v*) at 37 °C. Both solutions were mixed and stirred for 1 h, and subsequently, P_2_O_5_, CaP and SiO_2_ precursors solutions were added dropwise in intervals of 20 min. The product was collected after 24 h by centrifugation and calcined at 550 °C for 4 h.

PEI coating: Following the dispersion of 60 mg of nanoparticles in 20 mL of water, a 10 mL volume of 56 mM 3-(trihydroxysilyl) propylmethylphosphonate (THPMP) solution was added to the mixture and stirred at 40 °C for 2 h for surface phosphonate modification. The products were collected by centrifugation and resuspended in a PEI solution which was prepared by mixing 150 mg of PEI (10 kD) with 15 mL of 100 mM carbonate buffer (pH 9.6). The suspension was stirred at room temperature for 4 h. Finally, after centrifugation, the PEI-coated nanoparticles were washed and dried at room temperature.

### 2.2. Characterization of Nanoparticles

The size and morphology of nanoparticles were observed using scanning electron microscopy (SEM) (MIRA III, Tescan, Brno, Czech Republic), transmission electron microscope [42] (model HT7700, Hitachi, Tokyo, Japan), and dynamic light scattering (DLS). Nanoparticles were degassed overnight at 110 °C using a vacuum line. Then, the pore size distribution was specified from the N_2_ desorption branch of the obtained N_2_ absorption using a Micromeritics Tristar II porosity analyser system at −196 °C following the Barrett-Joyner-Halenda (BJH) method. The total pore volume was calculated based on the adsorbed amount, at maximum relative pressure (P/P0) of 0.99. The specific surface areas were calculated by Brunauer-Emmett-Teller (BET) method. The surface electrical potential (Zeta potential) was measured by a Zetasizer Nano-ZS (Malvern Instruments, Worcestershire, UK) at 25 °C with the nanoparticles dispersed in water at room temperature with an applied field strength of 20 V/cm. Fourier transform infrared (FTIR) spectroscopy was carried out to determine the chemical properties of nanoparticles before and after coating, using an FTIR Nicolet iS20 spectrometer (ThermoFisher Scientific, Scoresby, Australia) equipped with a Goldengate attenuated total reflectance device. In addition, energy dispersive analysis (EDX) was performed to assess the particle composition for MBGN. X-ray photoelectron spectroscopy (XPS) analysis has been performed to analyse the surface chemistry of both particles using a Kratos Axis ULTRA X-ray Photoelectron Spectrometer incorporating a 165 mm hemispherical electron energy analyser. The incident radiation was Monochromatic Al Kα X-rays (1486.6 eV) at 150 W (15 kV, 10 mA). The vacuum-dried samples were scanned at an analyser pass energy of 160 eV over 1200–0 eV binding energy range with 1.0 eV steps and a dwell time of 100 ms for a wide survey and at 20 eV with 0.05 eV steps and 250 Ms dwell time for narrow high-resolution survey. Atomic concentrations were calculated using the CasaXPS version 2.3.14 software. Samples were run in duplicates, and one spot on each sample was analysed. 

### 2.3. Preparation of Nanoparticle-MicroRNA Complexes

MSN-PEI or MBGN-PEI (10, 20, or 40 μg) were incubated in 50 μL of media without serum with 30 pmol of carboxyfluorescein (6-FAM) labelled, rno-miRNA-26a-5 mimic or plain negative control (NC) miRNA for 15 min. All miRNA samples were purchased from GenePharma (GenePharma Co., Shanghai, China). The negative control miRNA used is a non-functional sequence of 23 nucleotides (5′-UUCAAGUAAUCCAGGAUAGGCUTT-3′). As the purpose of this study was to assess the delivery system’s biological performance, NC miRNA was employed for all biofunctionality tests in addition to a functional miRNA (based on our previous results [43]).

### 2.4. Assessment of miRNA Loading and Release

PEI coated nanoparticles at different concentrations (10, 15, 20, 30, 40, 60, and 120 μg/mL) were mixed with 30 pmol (212.7 ng) of miRNA in 1 mL of deionized water to determine an optimal mixing ratio for achieving a high loading efficiency of miRNA. The suspensions were kept at room temperature for 15 min to allow loading. Following centrifugation at 10,000 rpm for 2 min, the supernatants were collected, and the amount of unloaded miRNA was determined by a spectrophotometer (NanoDrop 2000, ThermoFisher Scientific, Wilmington, DE, USA). This was subtracted from the initial amount of miRNA, to estimate the amount that was loaded onto the nanoparticles.

To assess the release profile, the suspensions containing miRNA loaded nanoparticles and 1 mL of RNase-free TE buffer were stored at 37 °C for 4 days. The supernatant was obtained each day and assessed by spectrophotometry, up to the saturation point.

### 2.5. Cell Culture

BMSCs were harvested from the femoral bone marrow of 10 weeks old Sprague Dawley rats, as described previously [44]. This procedure was approved by the Animal Ethics Committee of The University of Queensland (ethic approval number: ANRFA/DENT/433/18). Briefly, the bones were collected and washed using phosphate-buffered saline (PBS). Bone marrow was aspirated from the bone and dispersed into Dulbecco’s modified Eagle’s medium (DMEM) (Life Technologies, Carlsbad, CA, USA). The collected cells were washed and centrifuged two times and finally resuspended in DMEM containing 10% fetal bovine serum (ThermoFisher Scientific Australia, Scoresby, Australia) and 1% penicillin and streptomycin, and then cultured at 37 °C in 5% CO_2_ in a humidified incubator. All cells used in this study were within three to five passages.

### 2.6. Cell Viability Tests

BMSCs were seeded in 96-well plates at a density of 5 × l0^4^ cells/mL and cultured in DMEM with 15% FBS and 1% penicillin-streptomycin for 24 h, before being exposed to MSN, MSN-PEI, MBGN, MBGN-PEI at different concentrations (5, 10, 20, 40, 80, 160, and 320 μg/mL). Untreated cells were used as a negative control. After 1, 3, and 7 days, 10 μL 3-(4,5-dimethylthiazol-2-yl)-2,5-diphenyltetrazolium bromide (MTT) (Sigma, St. Louis, MO, USA) (5 mg/mL) was added to each well, and the cells incubated at 37 °C in 5% CO_2_ for another 4 h. After incubation, the reaction was terminated by adding dimethyl sulfoxide (DMSO) (Roche, Basel, Switzerland), and the plates were shaken for 15 min in the dark. The optical absorbance was read by a microplate reader (Infinite, Tecan Trading AG, Männedorf, Switzerland) at a wavelength of 565 nm, and the percentage of viable cells was calculated.

### 2.7. Transfection Efficiency and Cellular Uptake

Known amounts (10, 20, and 40 µg/mL) of FAM-miRNA/MSN-PEIs or FAM-miRNA/MBGN-PEIs were used to transfect BMSCs (5 × l0^4^ per mL density) for 6 h. Transfection efficiency and cellular uptake were measured by confocal laser scanning microscopy (CLSM) and flow cytometry. The commercially available transfection reagent Lipofectamine 2000™ (Invitrogen™, Carlsbad, CA, USA) was used based on the manufacturer’s instructions as the positive control.

For CLSM observations, BMSCs were fixed for 30 min in 4% paraformaldehyde, after which the cells were permeabilized with 0.1% Triton X (J.T. Baker, Phillipsburg, NJ, USA) for 10 min, and washed three times in PBS. Nuclei were stained by DAPI (4′,6-diamidino-2-phenylindole) (D1306, ThermoFisher Scientific), and actin filaments (in the cytoskeleton) were stained by Phalloidin (Alexa Fluor^®^ 555, ThermoFisher Scientific) for 30 min. Lastly, the samples were mounted on the glass slides and examined by a confocal microscope (Nikon C2+, Nikon, Tokyo, Japan). The intracellular distribution of FAM-labelled miRNA in each group was revealed using 488 nm laser excitation.

For flow cytometry, after transfection, cells were trypsinized and washed with PBS. Following fixation of the samples with paraformaldehyde, the number of FAM positive cells was quantified using a flow cytometer (FACS Canto II, BD Biosciences, San Jose, CA, USA), with 500 cells per sample, using excitation at 488 nm. FlowJo software version 10.6.2 (FlowJo LLC, Ashland, OR, USA) was used to analyse flow cytometry data.

### 2.8. Quantitative Real-Time Polymerase Chain Reaction (qRT-PCR)

BMSCs were cultured in 6-well plates at a density of 10^6^ cells per well. After transfection the cells with 1. MSN-PEI, 2. MBGN-PEI, 3. NC-miRNA-MSN-PEI, 4. NC-miRNA-MBGN-PEI, 5. miRNA-26a-MSN-PEI, or 6. miRNA-26a-MBGN-PEI were incubated with osteogenic medium (DMEM supplemented with 10 mM β-glycerophosphate, 50 μM ascorbic acid and 100 nM dexamethasone). A group of cells cultured in osteogenic media without exposure to nanoparticles was used as a negative control.

For determining the osteogenic function of the nanocarriers, the relative levels of expression of five target genes [Runt-related transcription factor 2 (Runx-2), alkaline phosphatase (ALPL), collagen type 1 (Col1α1), osteocalcin (OCN), and osteopontin (OPN)] were measured using qRT-PCR. The primers used are listed in Table 1. Briefly, after 7 and 14 days, total RNA was isolated with trizol (Invitrogen™, ThermoFisher Scientific, Australia). The extracted RNA was measured using a NanoDrop spectrophotometer (Thermo Scientific NanoDrop Products, Wilmington, DE, USA). A 2-ng amount of total RNA in each sample was used to create cDNA using Superscript II reverse transcriptase (Invitrogen). Real-time PCR was done using the SYBR Green PCR Master Mix (Applied Biosystems, Warrington, Cheshire, UK). Amplification curves for the reactions were assessed using LightCycler Software^®^, version 3.5 (Roche Molecular Biochemicals, Basel, Switzerland). The comparative CT method was used for relative qualification, and relative gene expression (2^−ΔΔCT^) was determined and used to calculate fold-change differences between control and differentiated cultures using the Gene Globe Analysis application (http://www.qiagen.com/geneglobe accessed on 11 June 2022). Glyceraldehyde 3-phosphate dehydrogenase (GAPDH) was used as a housekeeping gene, and its mRNA level was used to normalize results for the target genes of interest. All reactions were performed in triplicate.

### 2.9. ALP Activity Assay

After transfection of the cells with 1. MSN-PEI, 2. MBGN-PEI, 3. NC-miRNA-MSN-PEI, 4. NC-miRNA-MBGN-PEI, 5. miRNA-26a-MSN-PEI, or 6. miRNA-26a-MBGN-PEI, they were cultured in osteogenic media for 7 and 14 days. Untreated cells which were incubated in the same media but not transfected were used as a negative control. At the two designated time points, BMSCs were harvested, and ALP activity was assessed (ALP kit, Abcam, Cambridge, UK). Light absorbance at 405 nm was measured by a microplate reader (Infinite, Tecan Trading AG, Männedorf, Switzerland). ALP activity was interpolated from absorbance values of a standard curve of known concentrations of calf intestinal alkaline phosphatase and expressed as μmol/min/mL.

### 2.10. Matrix Mineralization Assessment

After transfection of the cells with different groups (group 1. MSN-PEI, 2. MBGN-PEI, 3. NC-miRNA-MSN-PEI, 4. NC-miRNA-MBGN-PEI, 5. miRNA-26a-MSN-PEI, and 6. miRNA-26a-MBGN-PEI), the cells were cultured in osteogenic media for 21 days. Untreated cells which were incubated in the same media but not transfected were used as a negative control. At 21 days, the formation of mineralized matrix nodules was evaluated by Alizarin red staining (Sigma, St. Louis, MO, USA). Firstly, the cells were fixed in 4% paraformaldehyde solution for 30 min, then washed with distilled water and stained with 5% Alizarin red for 1 h at room temperature. Then, nodules in each sample were examined using an inverted microscope. In addition, the amount of mineralization was quantified by diluting the samples in acetic acid, and then recording the absorbance at 405 nm, as described by Gregory et al. [45].

### 2.11. Degradation Test

A 2 mg amount of PEI coated nanoparticles were immersed in 20 mL of PBS buffer at 37 °C and pH 7 (100 μg/mL). All specimens were prepared in triplicate and incubated at 37 °C under constant stirring. On days 1, 2, 3, and 4, 2 mL samples were obtained for degradation analysis. The Si ion concentration was measured by Inductively Coupled Plasma Optical Emission Spectroscopy (ICP-OES) and the particle morphology was observed by TEM analysis.

### 2.12. Statistical Analysis

Data analysis was conducted using Prism (GraphPad, La Jolla, CA, USA). Data for cell viability (in percentages), gene expression, and ALP activity were subjected to one-way analysis of variance (ANOVA), with post hoc Tukey’s tests. A *p*-value less than 0.05 was considered statistically significant.

## 3. Results and Discussion

### 3.1. Characterization of Nanoparticles and Prepared Complexes

**Figure 1:**
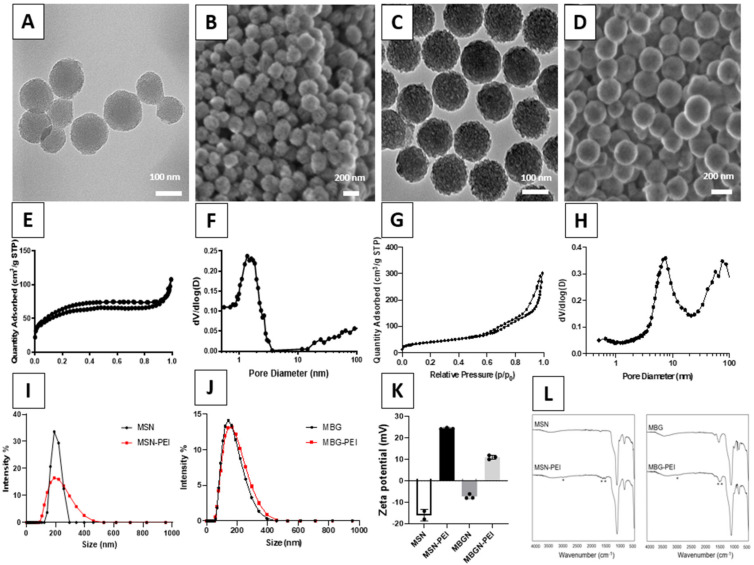
Characterization of MSN-PEI (panels **A**,**B**,**E**,**F**,**I**) and MBGN-PEI (panels **C**,**D**,**G**,**H**,**J**). TEM images (panels **A**,**C**), SEM images (panels **B**,**D**), and N2 sorption isotherms of MSN-PEI (**E**) and MBGN-PEI (**G**). Panels (**F**,**H**) are the pore size distribution curve of MSN-PEI and MBGN-PEI. Panels (**I**,**J**) show the particle size using dynamic light scattering analysis of MSN-PEI and MBGN-PEI. The Zeta (ζ) potential of particles before and after PEI coating is shown in panel (**K**). The Fourier-transform infrared spectroscopy (FTIR) is shown in panel (**L**) PEI (* and ** indicate the presence of new bands after functionalisation).

Figure 1 summarized the physicochemical characterization of MSN and MBGN with PEI coating. TEM images revealed porous structures in both nanoparticles (Figure 1A,C). SEM images showed a monodisperse distribution of spherical nanoparticles and both nanoparticles have a size of around 150 nm in diameter (Figure 1B,D). Nitrogen adsorption/desorption analysis showed that MSN-PEI follows type IV isotherm with a pore size of 1.4 nm (Figure 1E,F, Table 2). MBGN-PEI also showed a type IV isotherm with a large pore size (6.8 nm, Figure 1G,H, Table 2). The hydrodynamic sizes of both nanoparticles before and after PEI modification were tested by DLS. MSN showed a maximum size of around 200 nm. The size distribution curve of MSN-CC-PEI shifted to a little higher value after PEI functionalisation, becoming more homogeneous, with a slight decrease in the polydispersity index (0.369 ± 0.05 vs. 0.178 ± 0.02) (Figure 1I). MBGN-PEI showed fewer changes after coating in terms of the particle size distribution (polydispersity index: 0.209 ± 0.04 vs. 0.153 ± 0.02) (Figure 1J).

To confirm the PEI modification, the zeta potential of MSN and MBGN before and after modification was tested. For MSN, the zeta potential rose from −17.1 to +22.5 mV, due to the incorporation of positive charges (Figure 1K). Similar results were observed in MBGN, where the zeta potential increased from −7.8 to +9.7 mV. The PEI coating was also confirmed by FTIR spectroscopy (Figure 1L). The spectra of MSN and MBGN showed similar bands that are characteristic of Si-O bonds (between 490 and 1090 cm^−1^). MBGN showed bands at 1700 cm^−1^, which is probably due to Ca introduced in the silica network. After PEI coating, MSN-PEI and MBGN-PEI exhibited new bands at around 3800 cm^−1^ (N–H stretching), 3100–2800 cm^−1^ (C–H stretching) and 1600–1400 cm^−1^ (C-H and N–H vibrations of amino groups). In addition, after functionalization with PEI, the pore volume and BET surface area decreased for both types of nanoparticles (Table 2). Cationic polymeric coatings such as PEI which was used in this study attract negatively charged miRNA to the surface of nanoparticles via electrostatic interactions. In addition, PEI coating promotes the endosomal escape of miRNA into the cytosol [46] which is necessary for its function.

To further analyse the surface chemistry of both nanoparticles, we conducted an X-ray photoelectron spectroscopy (XPS) analysis for MSN-PEI and MBGN-PEI. Results showed there were O, N, C and Si elements on the surface of the MSN-PEI (Figure S1). For MBGN-PEI, in addition to the above 4 elements, a small peak of Ca be observed. We also quantified the element ratio using XPS. Results showed that MSN-PEI and MBGN-PEI had similar N ratios (10.63% vs. 8.53%). Since the N element was mainly from the PEI, this data indicated that those two nanoparticles had a comparable PEI amount after coating.

**Figure 2:**
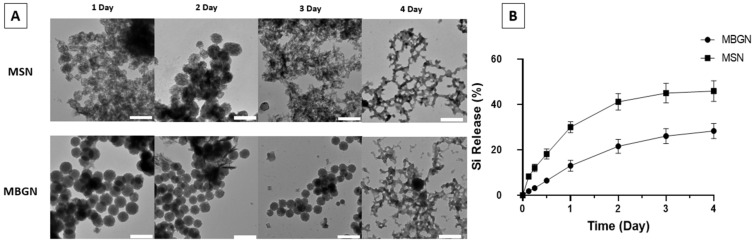
TEM images displaying the morphological changes of MSN-PEI and MBGN-PEI in PBS at pH 7 after 1, 2, 3 and 4 days (**A**) (scale bar 200 nm). The percentage of Si release was evaluated by ICP-OES (**B**).

Figure 2 shows the degradation of nanoparticles at near neutral pH (pH = 7.4, in PBS buffer) at 37 °C. Both MSN-PEI and MBGN-PEI kept their spherical morphology on days 1 and 2, but the pore structures were not as clear as as-synthesised ones. On days 3 and 4, the structure of MSN collapsed, which was consistent with the previous report [47]. For MBGN-PEI, the degradation was slightly slower compared to MSN-PEI and some MBGN-PEI were still spherical particles. On Day 4 most of the MBGN-PEI particles collapsed. The release profile tested by ICP-OES also showed the sustained release of Si ions from both MSN-PEI and MBGN-PEI and reached a plateau at day 4, which was consistent with the previous study [48].

### 3.2. miRNA Loading and Release

**Figure 3:**
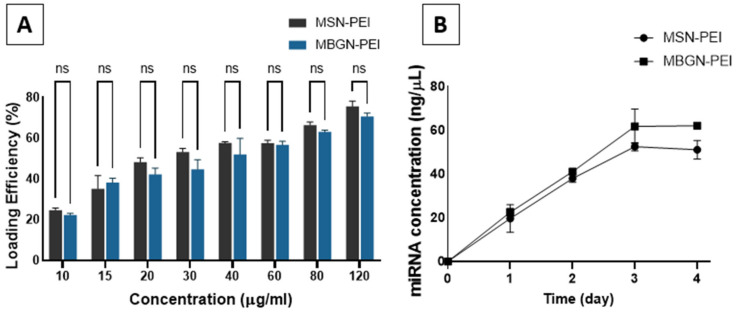
The loading efficiency (**A**) and the release profile (**B**) of MSN-PEI and MBGN-PEI for miRNA. A continual release for up to 3 days was observed for both groups with a final quantity of approximately 51% and 62% of the initial loading for MSN and MBGN groups, respectively, and then almost saturation. ns means there was no significant difference by post hoc Tukey tests (*n* = 3).

The loading efficiencies of miRNA onto MSN-PEI and MBGN-PEI were determined by comparing the amount of miRNA in the supernatant before and after adsorption (Figure 3A). The amount of miRNA loaded by both nanoparticles was 30 pmol (~0.6 µg miRNA). Loading efficiencies in both groups were dose-dependent, with the highest amount seen for 120 μg/mL for MSN-PEI and MBGN-PEI (75.7% ± 2.6 and 70.7% ± 1.6, respectively) (*p* = 0.95). One of the demerits of bioactive glass is its relatively lower loading gene efficiency. It has been shown that the gene loading efficiency of mesoporous bioactive glass could reach 20–50% in scaffolds [27]. Our results showed a significant improvement in the loading efficiency, which can be attributed to the porosity of the particles and PEI coating which enhanced the nucleic acid adsorption.

The release of miRNA from MSN-PEI and MBGN-PEI into PBS followed a linear pattern of increase for 3 days, which was slightly greater in the MBGN group, but not significantly (*p* > 0.05). The release amount saturated at 51% ± 4.3 and 62% ± 0.8 of the initial loading in the MSN and MBGN groups, respectively (*p* = 0.19) (Figure 3B). These findings indicate the loading efficiency and release of miRNA via the MSN-PEI and MGBN-PEI delivery systems are similar, and that they have the potential to release the miRNA cargo for at least 3 days. We determined the maximal loading quantity of miRNA onto MSN-PEI and MBGN-PEI and determined the optimal loading time in a pilot study (data not shown). The optimum loading time was 30 min.

### 3.3. Cell Viability

**Figure 4:**
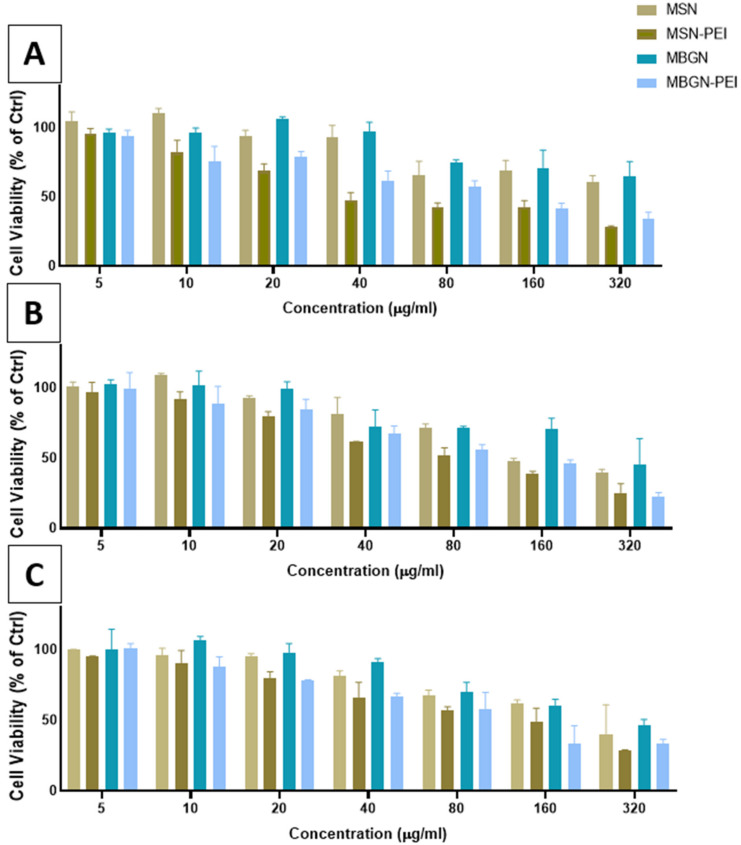
Cell toxicity after exposure to MSN and MBGN before and after coating with PEI. Data for MTT assay of rBMSCs after treatment with MSN, MSN-PEI, MBGN, and MBGN-PEI after 1 day (**A**), 3 (**B**), and 7 (**C**) days.

The percentages of viable BMSCs after incubation with various concentrations of MSN, MSN-PEI, MBGN, or MBGN-PEI were assessed using the MTT assay (Figure 4 panels A–C). The percentage of viable cells decreased in a concentrations dependant manner at 1, 3, and 7 days, for both PEI coated nanoparticles and uncoated nanoparticles. PEI coated particles showed considerably more cytotoxicity at doses higher than 40 μg/mL across 1, 3, and 7 days. In contrast, MSN and MBGN did not cause significant cytotoxicity, even at 160 μg/mL, across all time points. Overall, these results indicated that the safest conditions for the incubation of cells with the coated nanoparticles would be at concentrations of less than 20 μg/mL.

Cellular toxicity is known to be dependent on particle size, pore size and the surface chemistry of mesoporous nanoparticles [26]. Compared to MSNs, MBGNs showed similar or less toxicity. Overall, both nanoparticles showed high biocompatibility (>90% viability for doses less than 40 µg/mL). There were high percentages of viable BMSCs at very low doses (5, 10, 20 µg/mL). Although surface functionalization with PEI, as predicted, reduced the percentage of viable cells between 5 to 21% in a dose dependently manner, when used at a low dosage (i.e., less than 20 µg/mL) the PEI coated nanoparticles were associated with high cell viability (>75% after 7 days, Figure 4C).

### 3.4. Cellular Uptake and Transfection Efficiency

**Figure 5:**
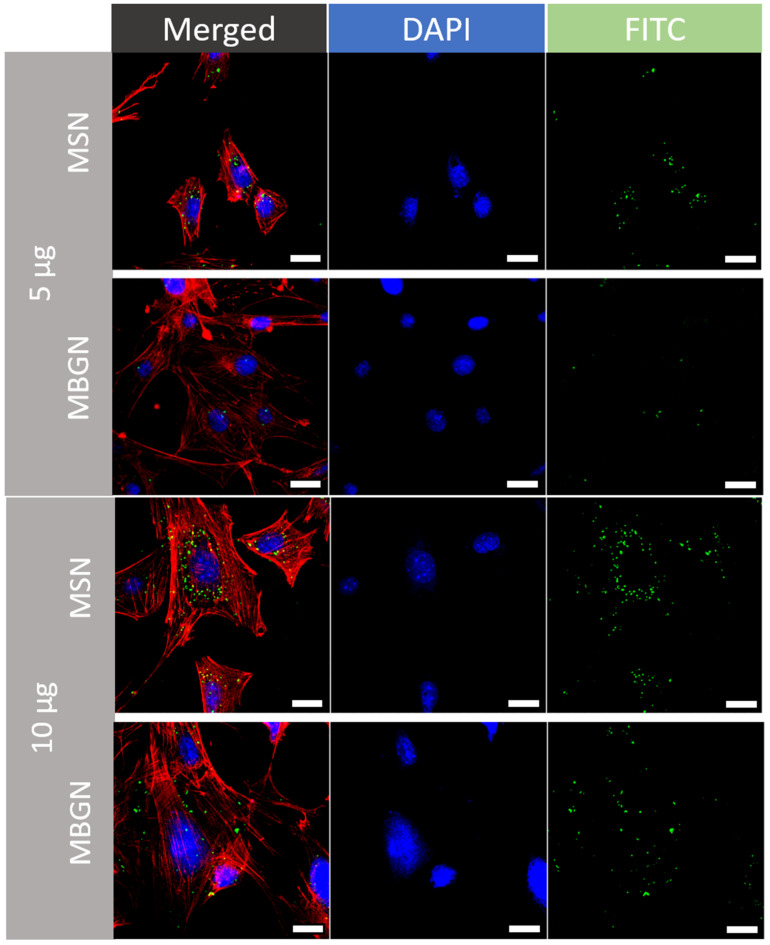

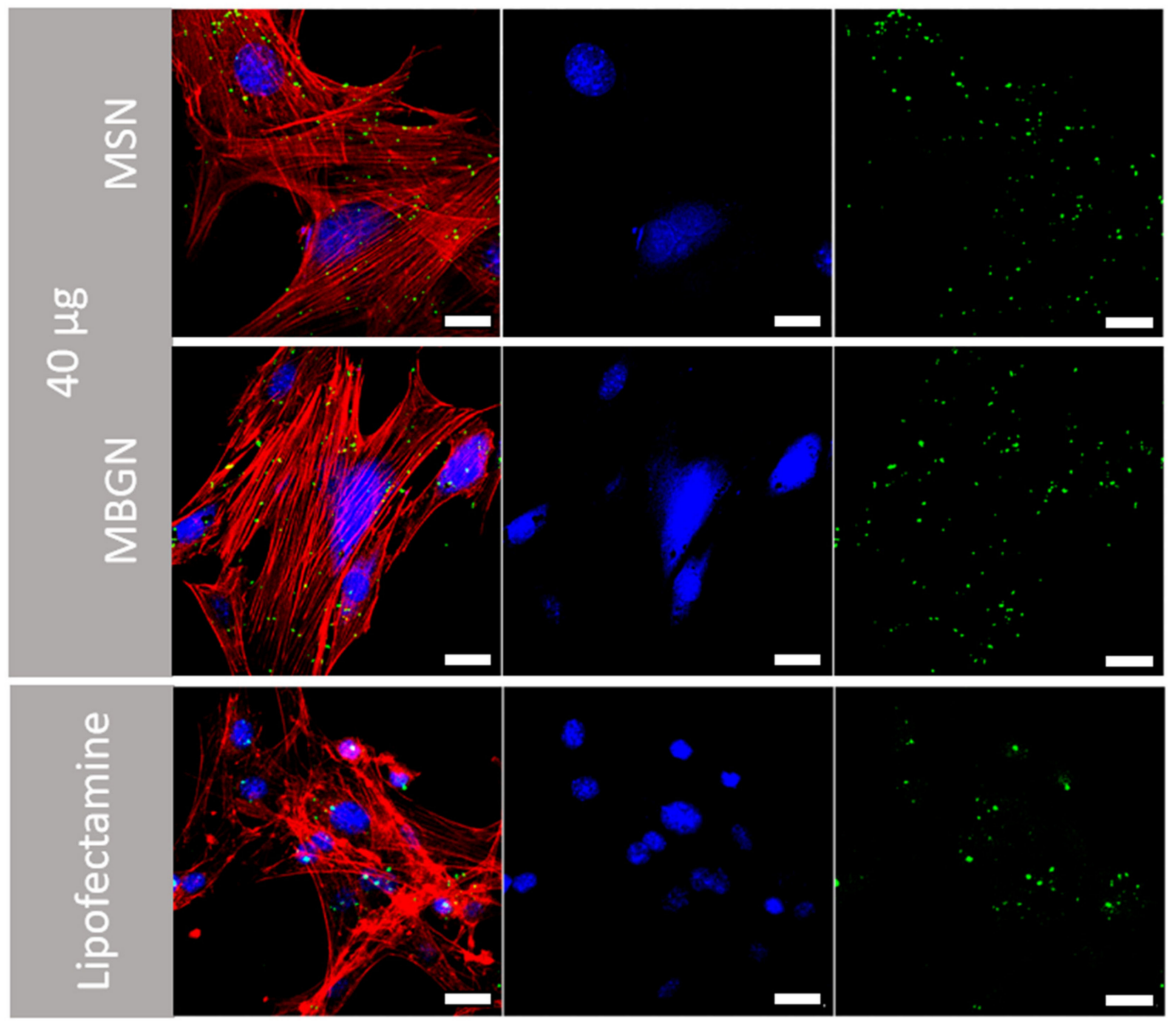
Transfection efficacy of FAM labelled microRNAs by MSN-PEI or MBGN-PEI towards rBMSCs. Representative confocal images of MSN-PEI and MBGN-PEI groups of rBMSCs after transfection with FAM labelled microRNAs (green) using nanoparticles for six hours. The cell nuclei were stained with DAPI (blue) and cell cytoskeletons were stained with Phalloidin (red) (Scale = 20 µm). Panel labels show the amount of nanoparticles (µg). Lipofectamine was used as a positive control.

Confocal microscope images of transfected BMSCs that had been incubated with 5, 10, or 20 µg of MSN-PEI or MBGN-PEI loaded with FAM labelled miRNA are shown in Figure 5. By 6 hr after incubation, green fluorescent dots were observed in the cytoplasm, as granular and concentrated areas, in both MSN and MBGN-treated groups. This indicates that FAM labelled miRNA loaded MSNs and MBGNs had been internalized by the BMSCs.

**Figure 6:**
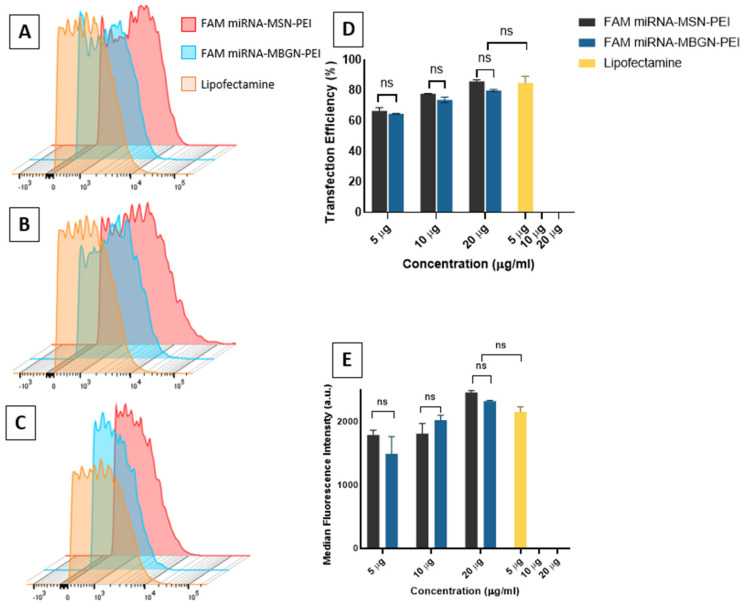
Transfection efficacy of FAM labelled microRNAs by MSN-PEI or MBGN-PEI towards rBMSCs. Stacked histogram of relative fluorescence intensity (**A**–**C**) of rBMSCs after transfection with FAM labelled microRNAs for six hours. The percentage of transfection efficiency (**D**) and the mean fluorescent intensity (**E**) (*n* = 3) were quantified with flow cytometry. Results are shown as mean ± SD. ns means there was no significant difference by post hoc Tukey tests (*n* = 3).

The results from CLSM revealed that cellular uptake increased as the dose of nanoparticles increased. Moreover, the flow-cytometric analysis confirmed the CLSM findings. The percentage transfection efficiency was not significantly different between the two nanoparticles in terms of the mean fluorescence intensity (Figure 6D) and the percentage of transfection efficiency (Figure 6E). The highest transfection efficiency in MSN-PEI and MBGN-PEI groups was achieved at a dose of 20 µg (85.8% ± 1.2 and 79.9% ± 0.7, respectively) (*p* = 0.73). The transfection efficiency of the lipofectamine group (positive control) was not significantly different in terms of the efficiency with both nanoparticles at 10 and 20 µg. Overall, these findings demonstrated the effectiveness of cellular uptake by both nanoparticle delivery systems.

MBGNs have recently been applied as gene delivery vectors [32,49,50]. Yu et al. demonstrated the intrinsic gene binding ability of MBGNs, due to their calcium ions. They showed that calcium ions interact with carboxyl, phosphate and sulphate groups in nucleic acids and that MBGNs without a coating have a transfection efficiency of approximately 45% [51]. This value can be improved. To do this, appropriate surface modifications of MBGNs are required, to present a positive charge on the surface and thus facilitate the nucleic acid complexation via electrostatic attractions. Amino-modification using 3-aminopropyltriethoxysilane (APTES) silanization is one of the frequently used methods [32,52]. The crossed-linked PEI as a cationic polymer allows the loading of negatively charged nucleic acids, as well as excellent endosomolytic activity via proton buffering effects [53,54]. Although it is a simple method, due to the lower positive charge on the surface, the amine modified-MBGN shows a lower gene loading capacity than MBGN with stronger basic functional groups such as guanidine or arginine [50]. In addition, the hydrophobic nature of alkyl amines may hamper biodegradation of the MBGN surface, a point which is undesirable for bone regenerative purposes.

Hence, new and potent surface modification methods are required to give MBGN optimized surface properties. In previous research, we showed that PEI coating is effective for MSNs as a silica-based nanovector [43]. MBGNs also have a large number of silanol groups on their surface, similar to MSNs, and these can be used as an initiating point for functionalization. In this study, the PEI coating provided superior cellular internalization and transfection efficiency for miRNA, for both MSNs and MBGNs. While amine MBGN achieved 53% of positive cells for small interfering RNA [32], the present results were approximately 86% and 80% positive cells, for MSN-PEI and MBGN-PEI, respectively. This strong performance is in accordance with other studies on PEI-coated MSNs [43,55,56,57,58], which demonstrated even with small (less than 5 nm) pores in the MSN, the highly positively charged surface (around +25 mV) allowed RNA complexation and resulted in excellent delivery to cancerous cells [56,58]. However, to our knowledge, the present study is the investigation where functionalized MBGN coated with PEI for miRNA delivery purposes has been compared to MSN-PEI, for use in bone regeneration.

### 3.5. ALP Activity and Gene Expression

**Figure 7:**
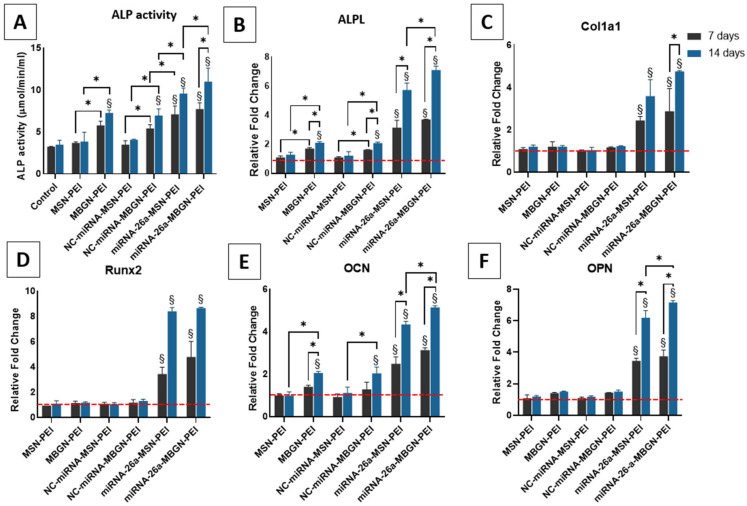
ALP activity assay (panel (**A**)) and gene expression (panels (**B**–**F**)) after treatment with MSN-PEI or MBGN-PEI loaded negative control (NC)-miRNA. Osteogenic-related mRNA expression after transfected with negative control (NC)-miRNA-MSN-PEI, MSN-PEI, NC-miRNA-MBGN-PEI, MBGN-PEI, or control for 7 (black bars) and 14 days (grey bars). Results are shown as mean ± SD. Red lines indicate the gene expression level from the blank control group. § *p* < 0.05 compared to the control group and * *p* < 0.05 compared to the experimental groups by post hoc Tukey tests (*n* = 3).

ALP is a marker of early osteogenesis [59], and its activity and gene expression were assessed 7 and 14 days after exposure to the nanoparticles with miRNA, as an indicator of osteogenic differentiation of BMSCs (Figure 7A). The functionality of the delivery system is further assessed by loading miRNA-26a which was previously evaluated by our research group and demonstrated high osteogenic activity [43]. The ALP activity and gene expression as expected for nanocomplexes with miRNA-26 were the highest among all groups which indicated the appropriate functionality of both nanoparticles. When MBGN-PEI was used for transferring miRNA-26a it showed higher values with a significant difference in ALPL expression after 14 days compared with miRNA-26a-MSN-PEI (7.1 ± 0.18 vs. 5.73 ± 0.34) (*p* > 0.05). Moreover, among non-functional and plain miRNA groups, the highest ALP activity was found after using MBGN-PEI with/without miRNA, therefore indicating a positive impact of MBGN-PEI on osteogenic differentiation (7.26 ± 0.33 μmol/min/mL for MBGN and 7.67 ± 0.50 μmol/min/mL for NC-miRNA-MBGN after 14 days). Moreover, this was confirmed by ALP gene expression at 14 days (Figure 7B). Overall, for ALP, there were no significant differences in terms of ALP activity and ALP gene expression between the MSN complexes (Plain or NC-miRNA) and control groups (*p* > 0.05). This indicates that MSN did not influence the osteogenic activity of BMSCs.

### 3.6. Gene Expression Profile

Bone formation is a dynamic and intricate procedure that involves a number of signalling systems including Wnt, Runx-2, bone morphogenetic proteins/Smads, Osterix, and hedgehog [60]. Many molecules activate different signalling pathways by paracrine or autocrine secretion, to regulate the expression of transcription factors. Recently, miRNAs have emerged as an important regulatory mechanism for controlling mesenchymal stem cells during bone development [61,62].

During the early phases of osteogenic differentiation, Runx-2 and ALP are crucial for the generation of mineralized tissue [59,63]. Col1α1, as the main constituent of the organic part of the extracellular matrix, is also commonly used as an early marker for osteogenic differentiation [64]. On the other hand, OCN and OPN as non-collagenous bone extracellular matrix proteins, are expressed mainly at the late stages of osteogenesis. Thus, in the present study, we examined the level of expression of these early and late osteogenic markers, after 7 and 14 days (Figure 7 panels C–F). MBGN-PEI/MSN-PEI loaded with miRNA-26a showed the highest results for all genes. In addition, MBGN-PEI with or without loaded miRNA significantly enhanced ALP and OCN expression after 14 days, compared to MSN and control groups (*p* < 0.05) (Figure 7 panels B,F). miRNA-26a-MBGN-PEI showed significantly higher expression of OCN after 14 days compared with MSN-PEI loaded by miRNA-26a (Figure 7E) (5.14 ± 0.05 vs. 4.34 ± 0.09) (*p* < 0.05). This indicates the application of MBGN even with the therapeutic agent allows higher expression of osteogenic markers. Although MBGNs also increased the expression of Runx-2 and OPN, the difference was not significant at any time point (Figure 7 panels D,F).

One of the important aspects of MSN as a nano-vector that is commonly investigated is its ability to incorporate nucleic acids and deliver them into cellular compartments. The mesoporous structure of these nanoparticles allows the loading of a large number of genes. Their small particle size allows for effective cellular uptake via endocytosis. The PEI coated MBGNs share these characteristics. Furthermore, this calcium containing MBGN has beneficial effects on the osteogenic differentiation of rBMSCs. This latter point is in agreement with previous reports of bioactive glass influencing bone cell functions such as stem cell differentiation and mineralization [65,66].

### 3.7. Mineralization Assay

**Figure 8:**
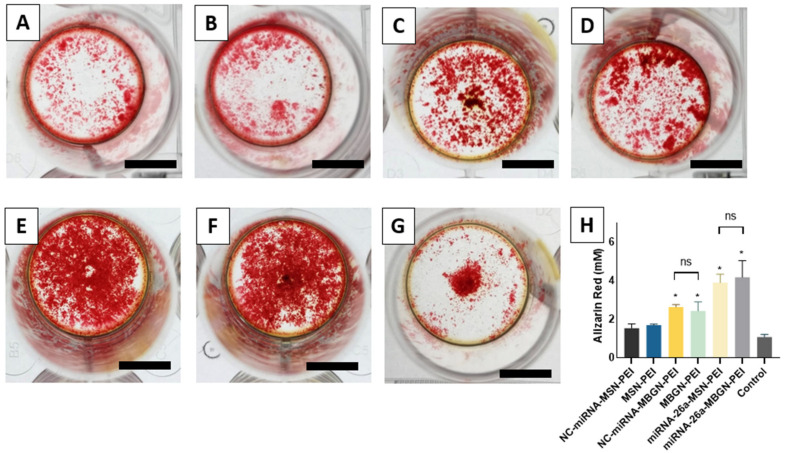
Mineralization and calcium secretion by BMSCs after 21 days of incubation. The cells were treated by transfected with negative control (NC)-miRNA-MSN-PEI (**A**), MSN-PEI (**B**), NC-miRNA-MBGN-PEI (**C**), MBGN-PEI (**D**), miRNA-26a-MSN-PEI (**E**), miRNA-26a-MBGN-PEI (**F**) or control (**G**) (bar = 1000 µm). Alizarin red staining was quantified by a spectrophotometer (**H**). * *p* < 0.05 compared to the control group by post hoc Tukey tests (*n* = 3).

Mineralization of the extracellular matrix was evaluated by Alizarin red staining at 21 days (Figure 8 panels A–G). Calcium deposition after transfection using MSN-PEI or MBGN-PEI loaded miRNA was significantly higher than controls (2.43 mM ± 0.47, 2.46 mM ± 0.12, and 1.08 mM ± 0.13, respectively) (*p* < 0.05) (Figure 8H). The highest values were observed in MBGN-PEI/MSN-PEI loaded with miRNA-26a which is in accordance with previous findings and support the functionality of both nanoparticles-based delivery system.

Taken together, the results for ALP activity, the expression of osteogenic genes, and matrix mineralization indicate that MBGN-PEI as a nanocarrier can also help drive osteogenesis, both in its early and later stages. This is consistent with the results of previous studies on bioactive glass [67]. MBGNs with their high surface bioreactivity and calcium ion release are helpful for bone regeneration [68,69]. PEI coating allows MBGN to be as effective as MSN in terms of gene transfection and cellular internalization. Additionally, MGBN-PEI showed successful delivery of a functional miRNA to boost osteogenesis. This opens a new attractive application for these silica-based nanoparticles as a gene delivery system for regenerative medicine.

## 4. Conclusions

Surface modified MBGN can serve as a mesoporous bioactive non-viral miRNA carrier to deliver miRAN as effectively as MSN. They can release miRNA continuously over 3 days and can transfect mesenchymal stem cells at rates as high as MSN (64–80%). Moreover, as a delivery system, MBGN also actively participates in the osteogenic differentiation of rBMSCs. They enhance ALP and OCN gene expression, and extracellular matrix mineralization. These findings together with high cell viability and high miRNA loading efficiency indicate that MBGN can be used as an efficient miRNA delivery system for osteogenesis.

## Figures and Tables

**Table 1:** Primer sequences used in this study for qRT-PCR assessment.

Gene Bank	Gene	Forward Reverse
NM_053470.1	RUNX2	5′-GAGCACAAACATGGCTGAGA-3′ 5′-TGGAGATGTTGCTCTGTTCG-3′
NM_013059.1	*ALPL*	5′-GCACAACATCAAGGACATCG-3′ 5′-TCAGTTCTGTTCTTGGGGTACAT-3′
NM_053304.1	Col1α1	5′-GCA ACA GTC GCT TCA CCT ACA-3′ 5′-CAA TGT CCA AGG GAG CCA CAT-3′
M25490.1	*OCN*	5′-TCTTTCTCCTTTGCCTGGC-3′ 5′-CACCGTCCTCAAATTCTCCC-3′
M14656.1	OPN	5′-CTGGCAGTGGTTTGCCTTTGCC-3′ 5′-CGTCAGATTCATCCGAGTTCAC-3′
NM_017008.4	GAPDH *	5′-TGTGTCCGTCGTGGATCTGA-3′ 5′-TTGCTGTTGAAGTCG CAGGAG-3′

* Housekeeping gene.

**Table 2:** Textural properties of MSN and MBGN before and after PEI coating.

Sample	Pore Size (nm)	Total Pore Volume (cm^3^/g)	BET Surface Area (m^2^/g)
MSN	1.94	0.21	242.27
MSN-PEI	1.37	0.16	214.13
MBGN	6.81	0.58	157.23
MBGN-PEI	6.81	0.45	140.25

## Supplementary Materials

The following supporting information can be downloaded at: https://www.mdpi.com/article/10.3390/pharmaceutics14112302/s1, Figure S1: The X-ray photoelectron spectroscopy (XPS) analysis of MSN-PEI (A) and MBGN-PEI (B). Figures on the right are the high-resolution scan of carbon and the element analysis.
