# Supplementary material for: A Comparative Study of Mesoporous Silica and Mesoporous Bioactive Glass Nanoparticles as Non-Viral MicroRNA Vectors for Osteogenesis
Source: Pharmaceutics. 2022 Oct 26;14(11):2302. doi: 10.3390/pharmaceutics14112302 (PMC9694756; doi:10.3390/pharmaceutics14112302)
Supplement: Supplementary file 1 [file pharmaceutics-14-02302-s001.zip › pharmaceutics-1916171-supplementary.pdf]

A

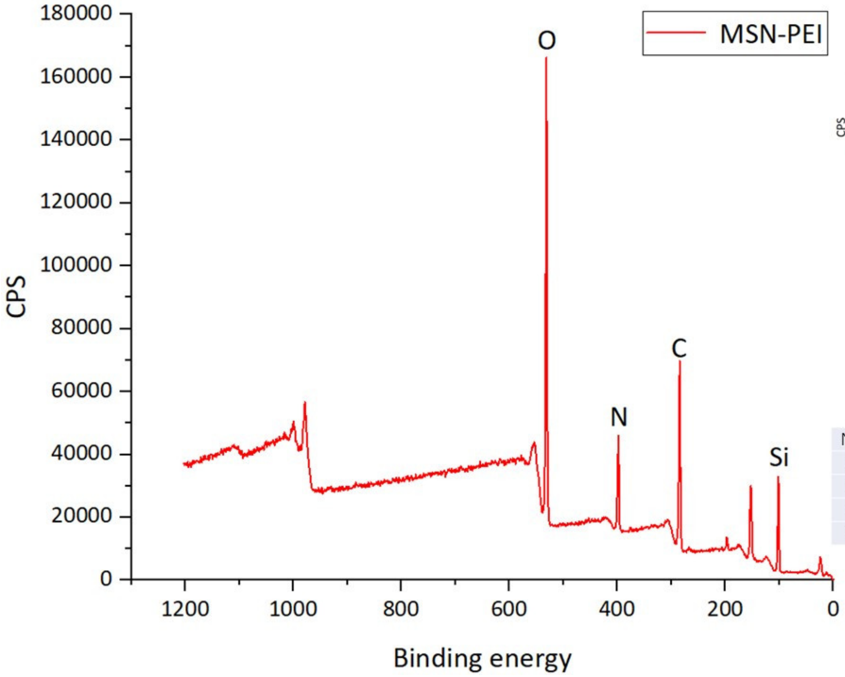

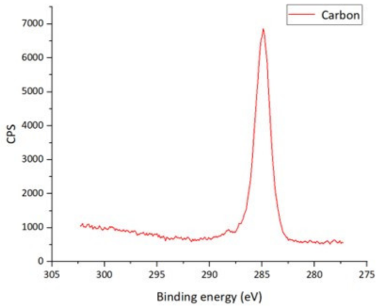

Name	Pos.	FWHM	Area	At%
C 1s	284.29	2.77	3616.6	38.07
N 1s	398.29	2.87	1817.24	10.63
O 1s	531.29	2.7	8351.84	30.01
Si 2p	102.29	2.72	1652.03	21.29

B

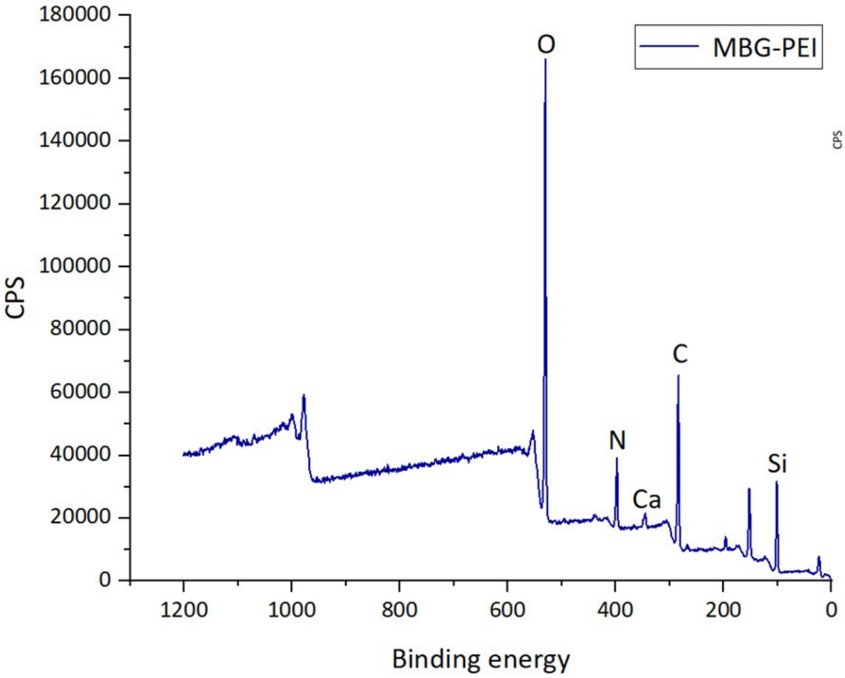

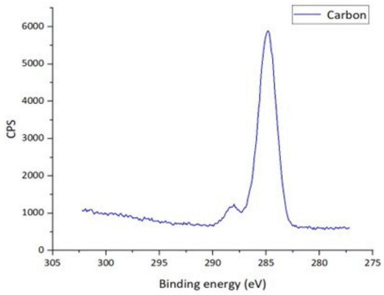

Name	Pos.	FWHM	Area	At%
C 1s	283.67	2.95	3582.2	38.37
N 1s	397.67	3.06	1433.9	8.53
O 1s	530.67	2.87	8716.6	31.87
Si 2p	101.67	2.75	1552.6	20.36
Ca 2p	345.67	5.53	411.98	0.87

Figure S1. The X-ray photoelectron spectroscopy (XPS) analysis of MSN-PEI (**A**) and MBGN-PEI (**B**). Figures on the right are the high-resolution scan of carbon and the element analysis.
